# Supplementary material for: Multiple Antibiotic Resistance (MAR), Plasmid Profiles, and DNA Polymorphisms among Vibrio vulnificus Isolates
Source: Antibiotics (Basel). 2019 May 28;8(2):68. doi: 10.3390/antibiotics8020068 (PMC6627359; doi:10.3390/antibiotics8020068)
Supplement: Supplementary file 1 [file antibiotics-08-00068-s001.pdf]

Table S1. Antibiotic resistance among *Vibrio vulnificus* isolates.

Isolate No.	Antibiotic Pattern [a]	Amp*	B*	Cpf	Da	E	K	Kf	Nv	P	S	T	Va	MR
1	BDaEKfNvPVa	1.3	-	1.7	-	-	2	-	-	-	1.6	2.2	-	7
2	BDaEKfNvPVa	1.2	-	2	-	-	2.2	-	-	-	1.8	2.3	-	7
3	AmpDaEKfNvP	-	1.2	1.5	-	-	1.7	-	-	-	1.5	1.3	1.2	6
4	BDaEKfNvPVa	1	-	2	-	-	2.5	-	-	-	1.8	2.5	-	7
5	AmpKfP	-	1.3	2.2	2	2	2	-	2.2	-	2	2	1.5	3
6	AmpBDaEKfNvPVa	-	-	1.8	-	-	2.5	-	-	-	2	1.5	-	8
7	BDaKfNvPVa	2	-	2.2	-	1.8	2.8	-	-	-	1.6	2.1	-	6
8	BDaKfNvPVa	1.3	-	1.8	-	1.8	2.5	-	-	-	1.8	2.5	-	6
9	AmpBDaKfNvPVa	-	-	2.5	-	2	2	-	-	-	2	2.6	-	7
10	AmpKfP	-	1.5	2.3	2	3	1.8	-	2	-	2	2	1.8	3
11	AmpKfP	-	2.1	1.6	2	2	1.3	-	1.2	-	1.2	2	1.8	3
12	PVa	1.3	1.8	2.8	2.5	2.2	2.2	1.6	2.5	-	2	2.3	-	2
13	AmpBDaEKfNvPVa	-	-	1.8	-	-	2.5	-	-	-	1.5	2	-	8
14	AmpBDaEKfNvPVa	-	-	2.5	-	-	3	-	-	-	2	2.5	-	8
15	AmpDaEKfNvP	-	1.7	2	-	-	2.5	-	-	-	1	2	1.6	6
16	AmpKfP	-	1.7	2	2.1	3.2	1.8	-	2	-	2.1	2.3	2	3
17	AmpKfP	-	1.3	1.8	1.8	3	1.3	-	2	-	1.8	2	2	3
18	AmpBDaKfNvPVa	-	-	1.5	-	1.7	2.2	-	-	-	1.5	2.5	-	7
19	AmpBDaEKfNvPVa	-	-	2	-	-	2	-	-	-	1.5	1.8	-	8
20	KfPVa	1.8	1.3	2.2	0.8	2.3	2.5	-	2	-	1.4	2.5	-	3
21	AmpBDaKfNvPVa	-	-	2.6	-	1.7	3	-	-	-	1.7	2.6	-	7
22	AmpBDaEKfNvPVa	-	-	1.8	-	-	2	-	-	-	1.1	2	-	8
23	AmpBDaEKfNvPVa	-	-	2.6	-	-	2	-	-	-	2	2	-	8
24	AmpPVa	-	2.3	2.3	2.3	3	2	1.1	2.2	-	2	2.8	-	3
25	AmpKfP	-	1.5	2.3	1.8	1.8	2.2	-	1	-	2	2	1.5	3
26	BDaEP	1	-	2.3	-	-	2.2	1.3	2.3	-	2	2.2	1.8	4
27	BDaENvPVa	1.5	-	2	-	-	1.8	2	-	-	1.8	2.3	-	6
28	AmpBDaE	-	-	2.2	-	-	2	1.5	2	1.5	2	2.5	1.8	4
29	AmpBDaEKfNvPVa	-	-	2.6	-	-	2	-	-	-	2	2	-	8
30	BDaEP	1.2	-	2.3	-	-	2.2	1.5	2.3	-	1.8	2.2	1.8	4
31	AmpPVa	-	2	2.5	2.3	3	2	1.5	2.2	-	2	2.8	-	3
32	AmpDaEKfNvP	-	1.2	2	-	-	1.7	-	-	-	1.5	2.3	1.5	6
33	BDaENvPVa	1.2	-	2	-	-	2.2	1.5	-	-	1.8	2.3	-	6
34	KfVa	1.5	2	2	1.5	2	2	-	1.5	1.3	2.3	1.8	-	2
35	AmpBDaEPVa	-	-	1.5	-	-	1.7	1.2	1.8	-	1.5	1.3	-	6
36	BDaEP	1	-	2.3	-	-	2.2	1.3	2.3	-	2	2.2	1.8	4
37	AmpDaEKfNvP	-	1.2	1.5	-	-	1.7	-	-	-	1.5	1.3	1.2	6
38	AmpBDaEKKfNvP	-	-	2	-	-	-	-	-	-	1.5	1.3	1.5	8
39	AmpKNvPVa	-	1.2	1.5	2	2.2	-	1.5	-	-	1.5	1.3	-	5
40	AmpDaEKfNvP	-	1.2	1.5	-	-	1.5	-	-	-	1.5	1.3	1.2	6
41	AmpDaKPVa	-	1.2	1.5	-	2	-	1.7	1.5	-	1.5	1.3	-	5
42	AmpPVa	-	2.3	2.3	2.3	3	2	1.1	2.2	-	2	2.8	-	3
43	AmpBDaEPVa	-	-	1.5	-	-	1.7	1.2	2	-	1.5	1.3	-	6
44	AmpPVa	-	2.3	2.3	2.3	3	2	1.1	2.2	-	2	2.2	-	3
45	AmpBDaEPVa	-	-	1.5	-	-	1.7	1.5	2.2	-	1.5	1.3	-	6
46	AmpKfP	-	1.5	2.3	1.8	1.8	2.2	-	1	-	2	2	1.5	3
47	AmpBDaKfNvPVa	-	-	1.8	-	2	2.5	-	-	-	1.5	2	-	8
48	AmpBDaEP	-	-	2	-	-	2.2	1.8	1.5	-	1.8	2.2	1.7	5
49	AmpKfP	-	1.5	2.3	1.8	1.8	2.2	-	1	-	2	2	1.5	3
50	AmpBDaEPVa	-	-	1.5	-	-	1.7	1.8	2	-	1.5	1.8	-	6
51	BDaEKfNvPVa	1.2	-	2	-	-	2	-	-	-	1.8	2	-	7
52	BDaEKfNvPVa	1.5	-	1.8	-	-	2.2	-	-	-	2	2.3	-	7
53	BDaEKfNvPVa	1.2	-	2	-	-	1.8	-	-	-	1.8	1.5	-	7

Isolate No.	Antibiotic Pattern [a]	Amp*	B*	Cpf	Da	E	K	Kf	Nv	P	S	T	Va	MR
54	AmpBDaEPVa	-	-	1.5	-	-	1.7	1.5	2	-	1.5	1.3	-	6
55	AmpBDaEPVa	-	-	1.5	-	-	1.7	1.8	1.5	-	1.5	1.3	-	6
56	AmpKfP	-	1.5	2.3	1.8	2	2.2	-	1	-	2	2	1.5	3
57	AmpKfP	-	2	2.3	1.8	1.8	2.2	-	1.5	-	2.3	2	1.5	3
58	KfVa	1.5	2	2	1.5	2	2	-	1.5	1.3	2.3	1.8	-	2
59	KfVa	1.3	1.8	2	2	2.2	1.8	-	1.8	1.3	2	1.5	-	2
60	BDaEKfNvPVa	1.2	-	2	-	-	2.2	-	-	-	1.8	2.3	-	7

[a]Tested for Ampicillin (Amp), Bacitracin (B), Cefoperazone (Cpf), Cephalothin (Kf), Clindamycin (Da), Erythromycin (E), Kanamycin(K), Novobiocin (Nv), Penicillin (P), Streptomycin (S), and Vancomycin (Va). MR- Multi resistance; - No diameter formed. Numbers- diameter in cm.

Table S2. Antibiotic resistance among *Vibrio vulnificus* isolates.

Isolate No.	antibiotic pattern [a]	Amp	B	Cpf	Da	E	K	Kf	Nv	P	S	T	Va	MR
1	BDaEKfNvPVa	S	R	S	R	R	S	R	R	R	S	S	R	7
2	BDaEKfNvPVa	S	R	S	R	R	S	R	R	R	S	S	R	7
3	AmpDaEKfNvP	R	S	S	R	R	S	R	R	R	S	S	S	6
4	BDaEKfNvPVa	S	R	S	R	R	S	R	R	R	S	S	R	7
5	AmpKfP	R	S	S	S	S	S	R	S	R	S	S	S	3
6	AmpBDaEKfNvPVa	R	R	S	R	R	S	R	R	R	S	S	R	8
7	BDaKfNvPVa	S	R	S	R	S	S	R	R	R	S	S	R	6
8	BDaKfNvPVa	S	R	S	R	S	S	R	R	R	S	S	R	6
9	AmpBDaKfNvPVa	R	R	S	R	S	S	R	R	R	S	S	R	7
10	AmpKfP	R	S	S	S	S	S	R	S	R	S	S	S	3
11	AmpKfP	R	S	S	S	S	S	R	S	R	S	S	S	3
12	PVa	S	S	S	S	S	S	S	S	R	S	S	R	2
13	AmpBDaEKfNvPVa	R	R	S	R	R	S	R	R	R	S	S	R	8
14	AmpBDaEKfNvPVa	R	R	S	R	R	S	R	R	R	S	S	R	8
15	AmpDaEKfNvP	R	S	S	R	R	S	R	R	R	S	S	S	6
16	AmpKfP	R	S	S	S	S	S	R	S	R	S	S	S	3
17	AmpKfP	R	S	S	S	S	S	R	S	R	S	S	S	3
18	AmpBDaKfNvPVa	R	R	S	R	S	S	R	R	R	S	S	R	7
19	AmpBDaEKfNvPVa	R	R	S	R	R	S	R	R	R	S	S	R	8
20	KfPVa	S	S	S	S	S	S	R	S	R	S	S	R	3
21	AmpBDaKfNvPVa	R	R	S	R	S	S	R	R	R	S	S	R	7
22	AmpBDaEKfNvPVa	R	R	S	R	S	S	R	R	R	S	S	R	8
23	AmpBDaEKfNvPVa	R	R	S	R	R	S	R	R	R	S	S	R	8
24	AmpPVa	R	S	S	S	S	S	S	S	R	S	S	R	3
25	AmpKfP	R	S	S	S	S	S	R	S	R	S	S	S	3
26	BDaEP	S	R	S	R	R	S	S	S	R	S	S	S	4
27	BDaENvPVa	S	R	S	R	R	S	S	R	R	S	S	R	6
28	AmpBDaE	R	R	S	R	R	S	S	S	S	S	S	S	4
29	AmpBDaEKfNvPVa	R	R	S	R	R	S	R	R	R	S	S	R	8
30	BDaEP	S	R	S	R	R	S	S	S	R	S	S	S	4
31	AmpPVa	R	S	S	S	S	S	S	S	R	S	S	R	3
32	AmpDaEKfNvP	R	S	S	R	R	S	R	R	R	S	S	S	6
33	BDaENvPVa	S	R	S	R	R	S	S	R	R	S	S	R	6
34	KfVa	S	S	S	S	S	S	R	S	S	S	S	R	2
35	AmpBDaEPVa	R	R	S	R	R	S	S	S	R	S	S	R	6
36	BDaEP	S	R	S	R	R	S	S	S	R	S	S	S	4
37	AmpDaEKfNvP	R	S	S	R	R	S	R	R	R	S	S	S	6
38	AmpBDaEKKfNvP	R	R	S	R	R	R	R	R	R	S	S	S	8
39	AmpKNvPVa	R	S	S	S	S	R	S	R	R	S	S	R	5
40	AmpDaEKfNvP	R	S	S	R	S	S	R	R	R	S	S	S	6

Isolate No.	antibiotic pattern [a]	Amp	B	Cpf	Da	E	K	Kf	Nv	P	S	T	Va	MR
41	AmpDaKPVa	R	S	S	R	S	R	S	S	R	S	S	R	5
42	AmpPVa	R	S	S	S	S	S	S	S	R	S	S	R	3
43	AmpBDaEPVa	R	R	S	R	R	S	S	S	R	S	S	R	6
44	AmpPVa	R	S	S	S	S	S	S	S	R	S	S	R	3
45	AmpBDaEPVa	R	R	S	R	R	S	S	S	R	S	S	R	6
46	AmpKfP	R	S	S	S	S	S	R	S	R	S	S	S	3
47	AmpBDaKfNvPVa	R	R	S	R	2	S	R	R	R	S	S	S	8
48	AmpBDaEP	R	R	S	R	R	S	S	S	R	S	S	S	5
49	AmpKfP	R	S	S	S	S	S	R	S	R	S	S	S	3
50	AmpBDaEPVa	R	R	S	R	R	S	S	S	R	S	S	R	6
51	BDaEKfNvPVa	S	R	S	R	R	S	R	R	R	S	S	R	7
52	BDaEKfNvPVa	S	R	S	R	R	S	R	R	R	S	S	R	7
53	BDaEKfNvPVa	S	R	S	R	R	S	R	R	R	S	S	R	7
54	AmpBDaEPVa	R	R	S	R	R	S	S	S	R	S	S	R	6
55	AmpBDaEPVa	R	R	S	R	R	S	S	S	R	S	S	R	6
56	AmpKfP	R	S	S	S	S	S	R	S	R	S	S	S	3
57	AmpKfP	R	S	S	S	S	S	R	S	R	S	S	S	3
58	KfVa	S	S	S	S	S	S	R	S	S	S	S	R	2
59	KfVa	S	S	S	S	S	S	R	S	S	S	S	R	2
60	BDaEKfNvPVa	S	R	S	R	R	S	R	R	R	S	S	R	7

[a]Tested for Ampicillin (Amp), Bacitracin (B), Cefoperazone (Cpf), Cephalothin (Kf), Clindamycin (Da), Erythromycin (E), Kanamycin(K), Novobiocin (Nv), Penicillin (P), Streptomycin (S), and Vancomycin (Va). S- sensitive, R-resistance; - No diameter formed.

Plasmid profiles: P1 P2 P2 - - P3 P4 - - P5 P3 P1 P6 - -

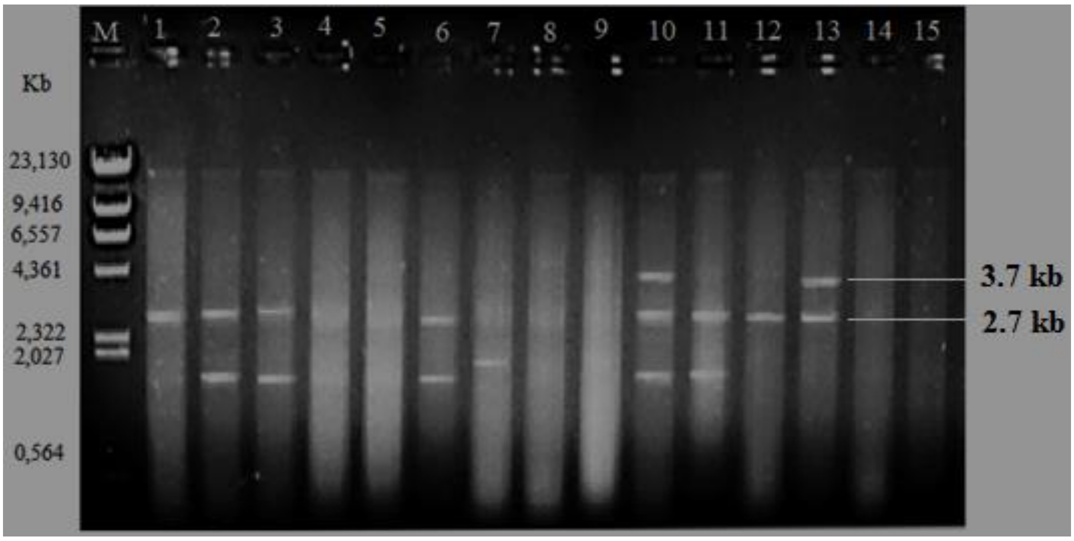

Figure S1. Plasmid identification using PureYield™ Plasmid Miniprep System kit (Promega, USA) on 1% (w/v) agarose gel. Lane 1: Lambda. DNA-HindIII Digest DNA ladder. Lane 1-15: Vv isolates 1-15.

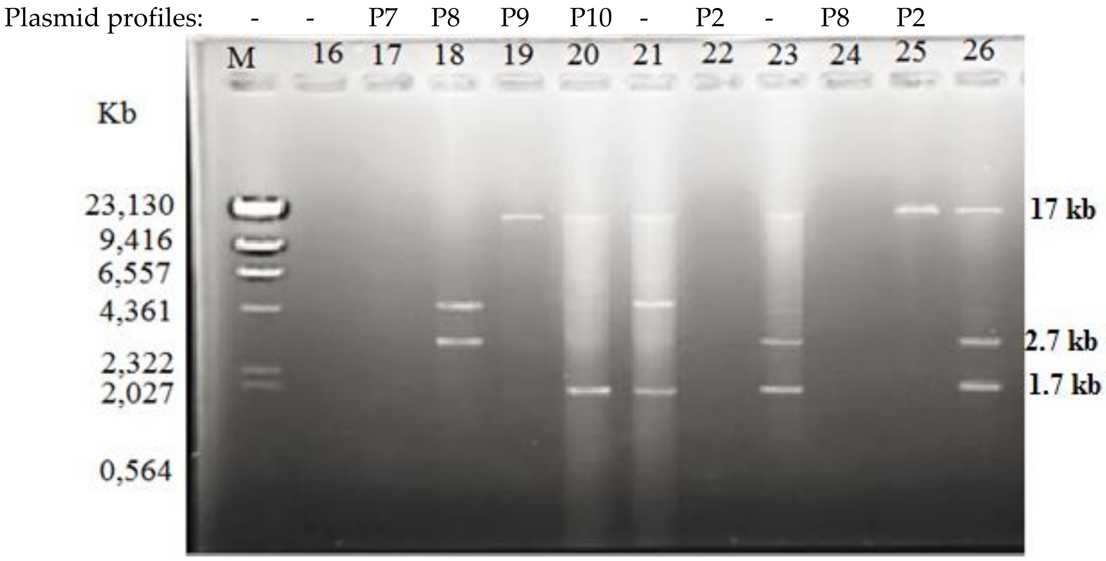

Figure S2. Plasmid identification using PureYield™ Plasmid Miniprep System kit (Promega, USA) on 1%(w/v) agarose gel. Lane 1: Lambda DNA-HindIII Digest DNA ladder. Lane 2-11: Vv isolates 16-26.

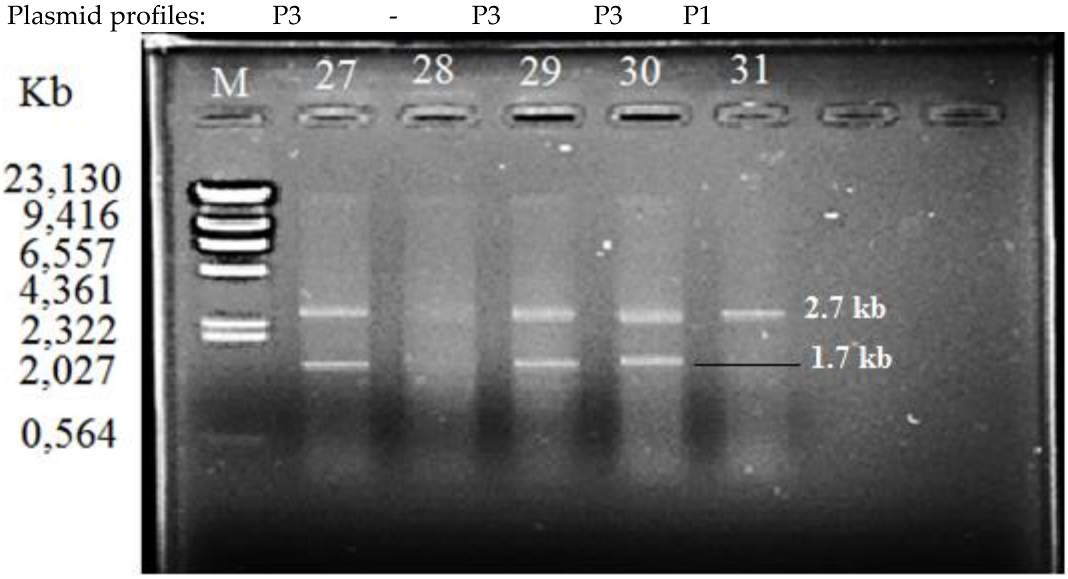

Figure S3. Plasmid identification using PureYield™ Plasmid Miniprep System kit (Promega, USA) on 1%(w/v) agarose gel. Lane 1: Lambda DNA-HindIII Digest DNA ladder. Lane 2- 6 Vv isolates 27-31.

Plasmid profiles: P11 P3 P3 - - P7 P12 P13 - - P14 - P3 P6 -

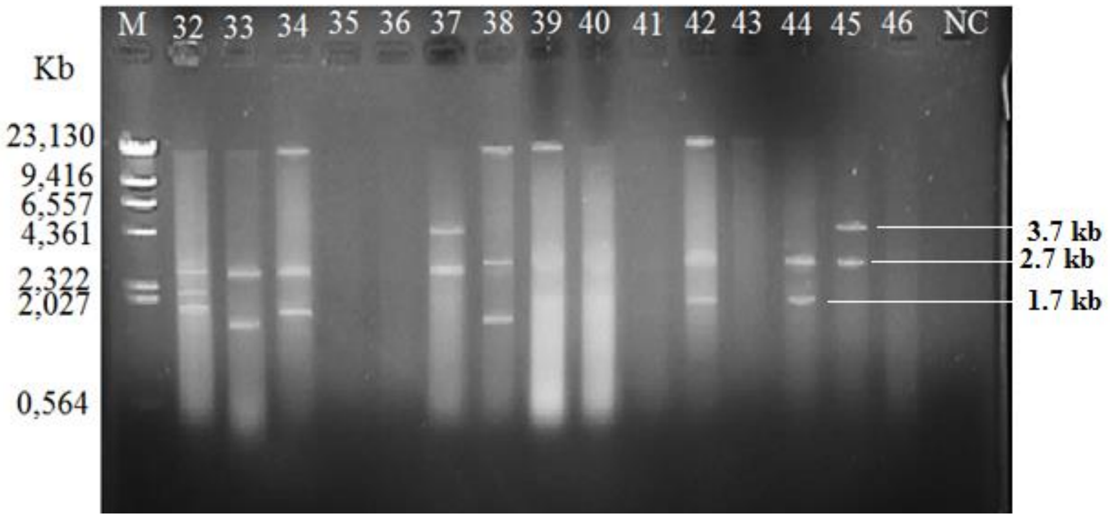

Figure S4. Plasmid identification using PureYield™ Plasmid Miniprep System kit (Promega, USA) on 1%(w/v) agarose gel. Lane 1: Lambda DNA-HindIII Digest DNA ladder. Lane 2-15: Vv isolates from 32 to 46 and lane 16: negative control.

Plasmid profiles: - P15 - - P16 P17 P18 P16 - P17 P18 - P19 P15

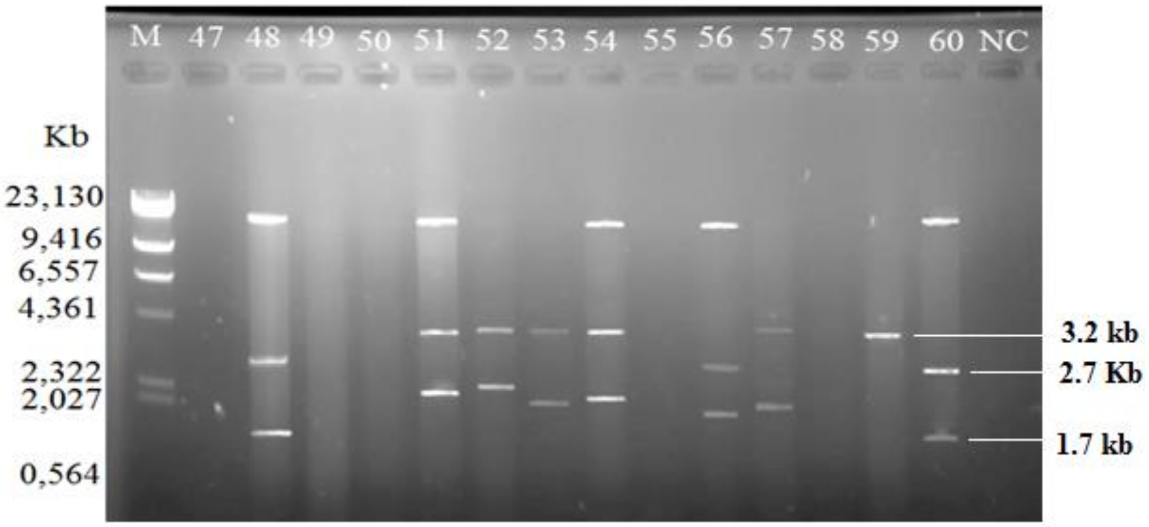

Figure S5. Plasmid identification using PureYield™ Plasmid Miniprep Systemkit (Promega, USA) on 1%(w/v) agarose gel. Lane M: Lambda DNA-HindIII Digest DNA ladder. Lane 2-15: Vv iolates from 47 to 60 and lane 16 NC: negative control.
